# Assessment of Nursing Faculty Members’ Knowledge Toward Research: A Cross-Sectional Study

**DOI:** 10.7759/cureus.62464

**Published:** 2024-06-16

**Authors:** Sirwan K Ahmed, Ribwar A Mohammed, Kochr A Mahmood, Omaid S Abdullah, Hazhar Blbas, Araz Q Abdalla, Mohammed K Hamad, Mona G Mohammed

**Affiliations:** 1 College of Nursing, University of Raparin, Ranya, IRQ; 2 Faculty of Medicine, Koya University, Koya, IRQ; 3 Department of Statistics, Salahaddin University-Erbil, Erbil, IRQ; 4 Department of Adult Health Nursing, RAK Medical and Health Sciences University, Ras Al-Khaimah, ARE

**Keywords:** nurse’s knowledge, research nurse, nursing faculty members, research method, nursing research, research, knowledge

## Abstract

Background and aim

In the context of contributing to healthcare and the well-being of a nation and its communities, nursing research plays a vital role in advancing the discipline of nursing science. Nursing research is essential for improving the quality of nursing care. This study aims to examine the research knowledge level of faculty members at the College of Nursing, University of Raparin, Kurdistan Region, Iraq.

Methods

We conducted a cross-sectional study involving 43 nursing faculty members at the University of Raparin. The survey was distributed using convenience sampling in April 2024. Statistical analysis was performed using IBM SPSS Statistics for Windows, Version 26.0 (released 2019, IBM Corp., Armonk, NY). Continuous variables were analyzed using mean and standard deviation, while categorical variables were analyzed using frequency and percentage distributions. The association between knowledge scores and demographics was tested using Mann-Whitney, Kruskal-Wallis, Chi-square, and post-hoc tests. Stepwise multiple regression analysis was used to determine the variables that influence the knowledge score. Significance was set at p < 0.05.

Results

A total of 43 participants were enrolled in this study. The mean age was 32.56 ± 4.75, with 69.8% of participants being male. In terms of educational attainment, 48.8% held master's degrees, 39.5% held bachelor's degrees, and only 11% possessed PhDs. The mean work experience was 5.40 ± 4.04 years, and the mean knowledge score toward research was 3.09 ± 1.97 out of 7. Demographic variables, such as age, marital status, and gender, showed no significant associations with knowledge levels (p > 0.05). However, significant associations were found for education level (p = 0.004) and years of experience (p = 0.011). In the stepwise regression analysis, we observed a significant positive correlation between the level of education and knowledge score (F =10.787, p = 0.002). However, variables, such as age, gender, marital status, years of experience, and participation in research courses, did not demonstrate statistical significance (p-values > 0.05).

Conclusion

This study highlights a significant lack of research expertise among nursing faculty members, emphasizing the urgent need for targeted interventions and instructional activities in nursing education programs. It is crucial to address this knowledge gap in order to support the professional growth of faculty members and promote the advancement of nursing research and evidence-based practice. Policymakers should also consider implementing mentorship programs that strategically pair less experienced faculty members with seasoned researchers. This initiative aims to cultivate a collaborative learning environment and enhance research skills.

## Introduction

Scientific research is a systematic investigation aimed at generating knowledge and understanding of different phenomena [1,2]. This process involves gathering data, documenting information, and then analyzing and interpreting the collected data using specific methods and procedures [3,4]. Educational institutions, including universities worldwide, are currently evaluated based on their scientific research, innovation, and knowledge outcomes [5]. Conducting research and sharing findings, especially within higher education institutions, is a crucial measure of one's contribution to the advancement of scientific knowledge and the development of innovative practices [6].

In the context of contributing to healthcare and the well-being of a nation and its communities, nursing research plays a vital role in advancing the discipline of nursing science [6,7]. It provides evidence-based practices that are essential for education and clinical decision-making [8]. Like other healthcare professionals in academic roles, nurses in academic positions are obligated to contribute significantly to the field through teaching, research, and community engagement [6]. Academic nurses, as leaders in the field, have the skills and opportunities to spearhead research efforts and make valuable contributions to nursing. However, clinical nurses, who are responsible for patient care and often have limited time, face challenges in engaging in research. Nevertheless, their contributions remain significant [9].

Research plays a vital role in nursing by driving evidence-based practice, promoting innovation, and improving patient care quality [10]. Faculty members in academic institutions have a crucial role in advancing nursing research and contributing to scholarly work [11]. Nursing research is pivotal for advancing nursing science and enhancing nursing care [12]. The primary objective is to improve patient care outcomes and recognize the value of nurses in the healthcare team [13]. Therefore, providing administrative support and comprehensive nursing education is essential to foster nurses' active participation in research activities and utilization of research findings in clinical practice [14]. Undergraduate nursing programs now include mandatory courses on nursing research to acknowledge its significance [12]. Papers authored by nurse scholars are highly regarded as tangible outcomes of scientific inquiry [15]. Thus, academic research production is an important benchmark for evaluating departments, institutions, and the strength and reputation of the nursing discipline. However, nursing lags behind other fields in this aspect [16]. There is a significant disparity in research output among faculty members in academia [17]. Therefore, it is necessary for academics to engage in research to generate knowledge and advance their careers. Various factors, such as group work, collaboration with foreign investigators, and mentoring, have been identified as contributors to enhancing research output among faculty members [16,17]. On the other hand, administrative tasks can impede productivity and limit research output among academics [18]. Traditionally, academic institutions primarily assessed faculty members' overall performance by evaluating their teaching ability [11]. However, there has been a shift in focus toward evaluating research performance, recognizing its importance in assessing the overall performance of faculty members and accurately acknowledging their research achievements [19].

Several studies have examined research knowledge. For instance, Aksoy et al. (2018) conducted a study in Turkey that explored the knowledge and attitudes toward clinical research among 306 nurses [15]. The findings revealed a moderate level of knowledge and a positive attitude toward clinical research. Likewise, a study conducted in Austria in 2011 among 1,825 graduate nurses found that 45.4% of nurses had a significant deficiency in research knowledge [10]. Furthermore, a study conducted in Iran in 2015 among 384 medical students demonstrated that the majority of participants possessed a favorable understanding of research [2].

Nursing research in the Kurdistan region faces unique challenges and opportunities due to its specific socio-political and healthcare landscape. Currently, the healthcare system is undergoing development, requiring the urgent application of evidence-based practices to improve patient care. However, there are significant obstacles to overcome, such as limited resources, political instability, and a shortage of experienced researchers. In addition, there are infrastructure deficiencies, including inadequate access to research facilities and technological tools, lack of time, and a lack of funding for research initiatives. Cultural factors can also hinder research efforts, as societal norms may influence the acceptance and implementation of new healthcare practices. Despite these challenges, there is an increasing recognition of the importance of nursing research, along with a motivated and youthful nursing workforce eager to contribute to healthcare advancement.

Currently, no studies have been conducted to gauge the level of research knowledge among nursing faculty members in the Kurdistan region of Iraq. This article aims to present a comprehensive investigation into the research knowledge of faculty members at the College of Nursing, University of Raparin, in their pursuit of research excellence. The significance of this analysis lies in its potential to offer valuable insights that can guide strategic initiatives aimed at cultivating a nurturing research environment. Such an environment can foster a culture of intellectual curiosity and academic excellence within the academic community.

## Materials and methods

Study design, sampling, time frame, setting, and participants

A cross-sectional study was conducted at the University of Raparin, in Ranya, Iraq, involving a total of 43 nursing faculty members. Data were collected through a survey using convenience sampling. All participating faculty members were employed at the university. The data were collected from April 20, 2024, to April 27, 2024. The study followed the Strengthening the Reporting of Observational Studies in Epidemiology (STROBE) checklist, which offers standardized guidelines for reporting cross-sectional studies [20].

Data collection tool, validity, reliability, and measures

The data were collected using a validated online-based, self-reported, structured questionnaire. The questionnaire was developed based on a previously published study [2], and the questions were modified to improve comprehension. To ensure accuracy and consistency, the questionnaire was drafted in English (see Appendix). In addition, the instrument was distributed to five nurses and statistics experts to obtain their feedback on the qualitative content validity. After evaluating and incorporating their comments, no items were removed. Before the main data collection period began, 10% of the study participants were recruited from the College of Nursing to assess the reliability of the questions. Then, a pilot study was conducted to evaluate the clarity of the questionnaire in advance. On the other hand, for the questionnaire's reliability, the researchers calculated the internal consistency coefficient. For the knowledge scale, Cronbach's alpha was 0.708, which is considered acceptable [21].

In the next phase of this study, data were collected from nursing faculty members at the University of Raparin. A total of 43 nursing faculty members agreed to participate in this study. The questionnaire consisted of two sections. The first section gathered demographic information on the faculty members of the Nursing College at the University of Raparin. This included their age, gender, marital status, level of education, participation in research publications and writing courses, and years of experience. The second section of the questionnaire comprised seven questions to assess the research knowledge among nursing faculty members at the University of Raparin. Each correct response was assigned a score of 1, while each incorrect answer received a score of 0. Scores within the 75% interquartile range indicated good knowledge.

Inclusion and exclusion criteria

The present study employed specific inclusion and exclusion criteria. To be eligible for participation, individuals needed to give informed consent and be currently employed at the College of Nursing, University of Raparin. Participants who did not complete the entire questionnaire or had missing variables were not included in the study.

Outcomes

The study's main findings center on faculty members' knowledge of research. The researchers used demographic factors, such as age, education, and years of experience as independent variables.

Ethical considerations and informed consent

On the survey's introductory page, a section was included to request informed consent. Before proceeding with the survey, all participants involved in the study provided written informed consent. The principles guiding this survey encompassed non-harm, confidentiality, and preservation of respondents' privacy. The study obtained approval from the Ethics Committee of the College of Nursing at the University of Raparin, with identification number 38, in April 2024.

Statistical analysis

To conduct the statistical analysis, we used IBM SPSS Statistics for Windows, version 26.0 (released 2012, IBM Corp., Armonk, NY). We computed the mean and standard deviation (SD) for continuous variables, such as age, years of work experience, and knowledge scores. For categorical variables, we used frequency and percentage distributions for analysis. Given that the data did not adhere to a normal distribution, we applied non-parametric tests. To evaluate the association between knowledge scores and demographic variables, we used the Mann-Whitney U, Kruskal-Wallis, chi-square tests, and stepwise multiple regression analysis to determine the variables that influence the knowledge score. We considered a p-value of <0.05 as statistically significant.

## Results

Out of the 53 nursing faculty members at the University of Raparin who agreed to participate in this research, a total of 43 faculty members submitted their responses and completed the questionnaire, resulting in a response rate of 81.13%. Table 1 provides an overview of the demographic information of the 43 academic nursing faculty members from the College of Nursing at the University of Raparin who took part in the study. The mean age of the participants was 32.56 ± 4.75. The majority of the participants were male, accounting for 69.8% of the total sample. Furthermore, 53.5% of the participants were married. In terms of educational attainment, it was observed that 48.8% of the members held master's degrees, while a smaller proportion had bachelor's degrees (39.5%). Only 11% of the respondents held PhD degrees (Table 1). 

**Table 1:** Descriptive statistics for the demographic respondents.

Variables		Frequency (n)	Percentage (%)
Age group (years)	<=30	19	44.2
	31+	24	55.8
Gender	Female	13	30.2
	Male	30	69.8
Marital status	Single	20	46.5
	Married	23	53.5
What is your current degree?	Bachelor	17	39.5
	Master	21	48.8
	PhD	5	11.6
Experience group	1-3	19	44.2
	4-6	10	23.3
	7+	14	32.5

The study findings showed that the mean duration of work experience for faculty members was 5.40 ± 4.04 years. In addition, the mean knowledge score toward research among nursing faculty members at the University of Raparin was found to be 3.09 ± 1.97 out of 7, as shown in Table 2.

**Table 2:** Descriptive statistics for age, years of experience, and knowledge scores toward research.

Descriptive statistics	Age	Year of experience as faculty member	Knowledge scores
Mean	32.56	5.40	3.09
Median	31.00	4.00	3.00
Mode	30	2	5
Std. deviation	4.757	4.048	1.974
Variance	22.633	16.388	3.896
Minimum	24	0	0
Maximum	45	15	6

Table 3 presents the different levels of expertise demonstrated by participants when responding to important research-related inquiries. It is important to note that the participants showed a higher level of accuracy in identifying the software used for citing scientific papers, with 65.1% providing correct responses. However, challenges were observed in other areas, such as determining the range of blood types (with an accuracy rate of 51.2%) and adhering to the proper format for citing references in general medical dissertations and most medical journals (with a correctness rate of 25.6%). In addition, there was a significant lack of understanding of the definition of Medline, with only 37.2% of the participants answering accurately. Similarly, only 39.5% of the respondents correctly identified the section of an article that discusses study limitations. Interestingly, there was a noticeable discrepancy in defining the specific type of research that commonly experiences sample loss, as an unexpected 86.0% of participants provided inaccurate responses. Lastly, the participants were assessed on their knowledge of items that are not typically found in a scientific original article. Out of all the participants, 74.4% correctly identified these inappropriate items (Table 3).

**Table 3:** Descriptive statistics for knowledge questions toward research among nursing faculty members.

Questions	Incorrect answer		Correct answer	
	n	%	n	%
Which following software is used for reference of science article?	15	34.9%	28	65.1%
What is the variable scale of blood type?	21	48.8%	22	51.2%
Which way of writing reference is approved and for writing general medical dissertations and most medical journals?	32	74.4%	11	25.6%
What is the definition of Medline?	27	62.8%	16	37.2%
In which part of an article do you talk about the study limitations?	26	60.5%	17	39.5%
Which of the following types of research sees sample loss more commonly?	37	86.0%	6	14.0%
Which item is not part of a scientific original paper?	11	25.6%	32	74.4%

**Figure 1:**
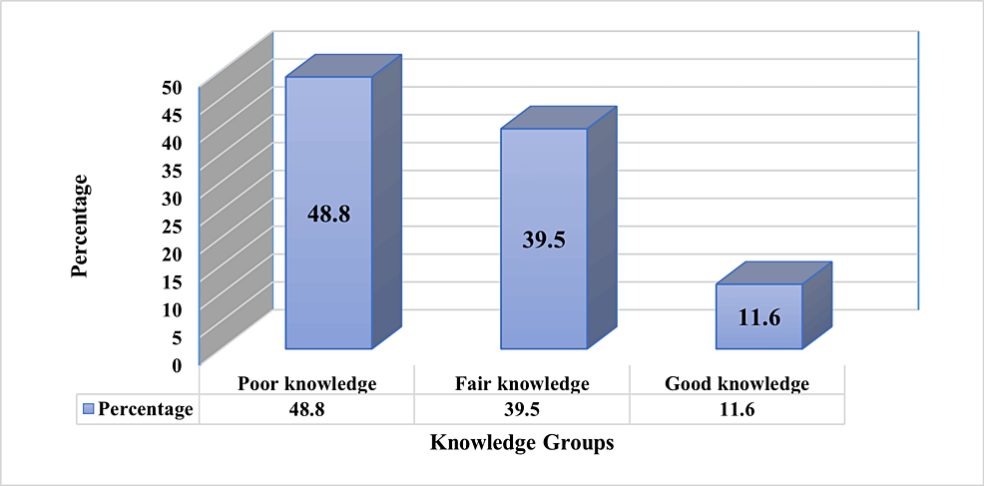
Level of knowledge toward research among nursing faculty members.

Figure 1 illustrates the distribution of participants based on their knowledge levels. It is evident that a considerable portion of nursing faculty members; specifically, 48.8%, had poor knowledge. In addition, 39.5% of the participants demonstrated fair knowledge. A smaller percentage, 11.6%, exhibited a commendable understanding of research (Figure 1).

Table 4 presents the analysis of demographic variables and shows interesting trends in knowledge levels among the participants. The results indicate that there was no substantial correlation between age groups and knowledge levels (χ² = 4.587, p = 0.101). Marital status also did not significantly influence the distribution of knowledge (χ² = 5.304, p = 0.071). Similarly, gender differences were not found to have a statistically significant impact on knowledge levels (χ² = 3.778, p = 0.151). However, there was a significant association between education levels and knowledge levels (χ² = 15.579, p = 0.004), suggesting variations in knowledge across different educational backgrounds. In addition, years of experience had a significant effect on knowledge levels (χ² = 13.111, p = 0.011), highlighting the influence of professional experience on the acquisition of knowledge toward research (Table 4).

**Table 4:** Association between demographic variables and knowledge groups toward research. χ² = chi-square test

Demographic variables			Knowledge group			χ²	p-value
			Poor knowledge	Fair knowledge	Good knowledge		
Age	<=30	Count	11	8	0	4.587	0.101
		%	57.9%	42.1%	0.0%		
	31+	Count	10	9	5		
		%	41.7%	37.5%	20.8%		
Marital status	Single	Count	12	8	0	5.304	0.071
		%	60%	40%	0.0%		
	Married	Count	9	9	5		
		%	39.1%	39.1%	21.7%		
Gender	Female	Count	4	8	1	3.778	0.151
		%	30.8%	61.5%	7.7%		
	Male	Count	17	9	4		
		%	56.7%	30.0%	13.3%		
Level of education	BSc	Count	11	6	0	15.579	0.004
		%	64.7%	35.3%	0.0%		
	MSc	Count	10	9	2		
		%	47.6%	42.9%	9.5%		
	PhD	Count	0	2	3		
		%	0.0%	40%	60%		
Years of experience	1-3	Count	12	6	0	13.111	0.011
		%	66.7%	33.3%	0.0%		
	4-6	Count	7	2	1		
		%	70%	20%	10%		
	7+	Count	2	8	4		
		%	14.3%	57.1%	28.6%		

The results of the Mann-Whitney U tests, which examined the variations in knowledge scores across different demographic variables, are shown in Table 5. Participants aged 31 and above had a higher average knowledge score (M = 3.667, mean rank = 25.90) compared to younger participants (M = 2.316, mean rank = 17.08) (Z = -2.318, p = 0.020). In terms of gender, although females had a slight numerical advantage (M = 3.769, mean rank = 28.23) over males (M = 2.767, mean rank = 20.17), the Mann-Whitney U test did not establish any statistical significance (Z = -1.475, p = 0.140). However, a statistically significant difference in knowledge scores was observed between married and single individuals. Married participants demonstrated significantly higher scores (M = 3.696, mean rank = 26.04) compared to single individuals (M = 2.350, mean rank = 17.35), as evidenced by a standardized test statistic (Z = -2.296, p = 0.022) (Table 5).

**Table 5:** Difference between the mean of knowledge score toward research with each of the (age group, gender, and marital status).

Demographic variables		n	Mean	Mean rank	Standardized test statistic (Z)	p-value
Age	<=30	19	2.316	17.08	-2.318	0.020
	31+	24	3.667	25.90		
Gender	Female	13	3.769	28.23	-1.475	0.140
	Male	30	2.767	20.17		
Marital status	Single	20	2.350	17.35	-2.296	0.022
	Married	23	3.696	26.04		

The analysis of knowledge scores across different levels of education revealed statistically significant differences among the groups. Participants with a bachelor's degree had a mean score of 2.294 and a mean rank of 17.15. Similarly, those with a master's degree had a higher mean score of 3.143 and a mean rank of 22.31. On the other hand, participants holding a PhD demonstrated the highest mean score of 5.400 and a mean rank of 37.20. The chi-square test yielded a value of 10.154, with a p-value of 0.006 (Table 6).

**Table 6:** Difference between the mean of knowledge score toward research and level of education.

Level of education	n	Mean	Mean rank	Chi-square test	p-value	
						Bachelor	17	2.294	17.15	10.154	0.006	
Master	21	3.143	22.31								
PhD	5	5.400	37.20								

**Figure 2:**
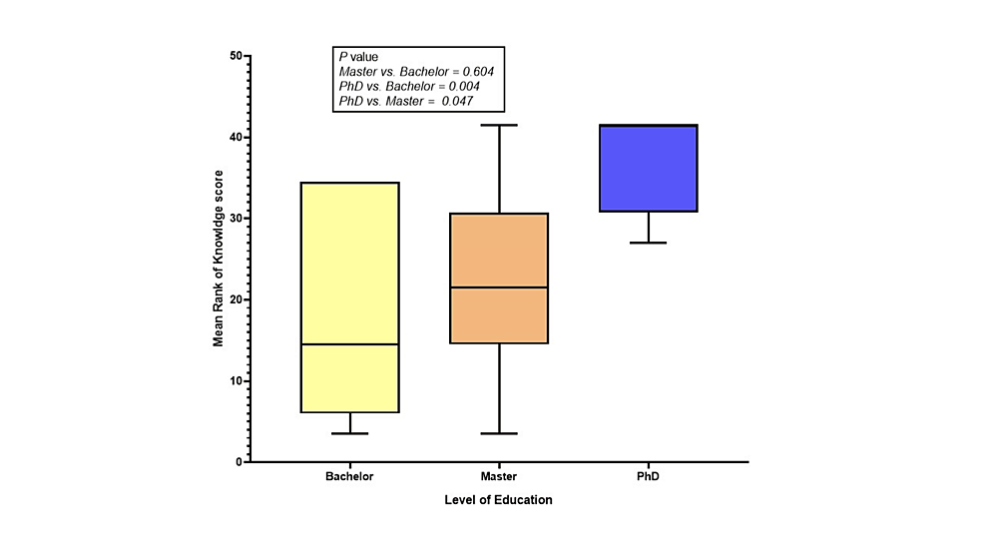
Association level of education in terms of knowledge score toward research. The post-hoc test revealed that individuals with a PhD had a higher mean rank of knowledge scores toward research compared to those with master's and bachelor's degrees.

Figure 2 shows the association between research knowledge and educational attainment using post-hoc tests. The analysis indicates that there is no significant association between individuals with a master's degree and those with a bachelor's degree (P = 0.604). However, individuals with a PhD have a significantly higher level of research knowledge compared to both master's and bachelor's degree holders (P = 0.047 and P = 0.004 ), respectively (Figure 2).

Table 7 demonstrates a significant positive correlation between the level of education and the knowledge score. The results of the ANOVA indicate that the model fits well, with the level of education significantly predicting the knowledge score (F = 10.787, P = 0.002). The regression coefficient (B) for the level of education is 1.332, suggesting that a one-unit increase in the level of education leads to a 1.332 increase in the knowledge score. The R-square value of 0.208 indicates that 20.8% of the variation in the knowledge score is explained by the level of education. Other independent variables (age, gender, marital status, years of experience, and participation in research writing and publication courses) were not found to have a statistically significant effect on the knowledge score (p-values > 0.05) (Table 7).

**Table 7:** Stepwise multiple regression analysis between independent variables (age, gender, marital status, level of education, years of experience, and participation in research writing and publication courses) and dependent variable (knowledge score).

Model	Coefficients				Model summary			ANOVA	
	B	t	P-value	Correlation		R Square	F		P-value
Constant	2.110	5.329	0.000	0.456		0.208	10.787		0.002
Level of education	1.332	3.284	0.002						

## Discussion

Nursing research is crucial in advancing nursing science and has a significant impact on healthcare and the well-being of nations and communities. With this in mind, our study aimed to assess the level of scientific research knowledge among the faculty members of the nursing college at Reparin University in Iraq. It is evident that a considerable portion of nursing faculty members, specifically 48.8%, had poor knowledge. In addition, 39.5% of the participants demonstrated fair knowledge. A smaller percentage, 11.6%, exhibited a commendable understanding of research

An examination of the educational credentials of the faculty members revealed that nearly half had successfully completed master's degrees, indicating that a significant number of individuals had advanced education. In addition, over a third of respondents held bachelor's degrees, showing a substantial presence of faculty members with undergraduate qualifications. By contrast, only a relatively small percentage of participants (11%) had obtained PhD degrees, suggesting a lesser prevalence of doctoral-level education among the surveyed faculty members. On average, the faculty members reported a mean work experience duration of 5.40 years, with a standard deviation of 4.04 years, indicating a moderate level of experience within the group. The combination of diverse educational backgrounds and experience levels has the potential to facilitate a wide range of research interests and methodologies. However, strategic initiatives may be necessary to enhance the research capabilities of the faculty members, such as providing opportunities for professional development, encouraging collaborations with researchers holding PhD degrees, and offering incentives for further education [22]. Regarding the faculty members' knowledge in various domains, it was observed that nearly two-thirds displayed proficiency in using and differentiating between software tools for citing scientific papers. However, many struggled with adhering to the correct format for citing references. In addition, there was a significant deficit in understanding the definition of Medline, with only one-third providing accurate responses. Similarly, slightly over one-third correctly identified the section of an article that discusses study limitations. A notable disparity emerged in the correct definition of the specific type of research that frequently experiences sample loss, with an unexpected majority providing inaccurate answers. Finally, the participants were assessed on their familiarity with items that are not commonly encountered in a scientific original article. Two-thirds of the participants accurately identified the items that were deemed inappropriate.

Deficiencies in research knowledge can significantly influence the quality of nursing research. A prime illustration of this is the inadequate understanding of how to effectively navigate databases, such as Medline. As a result, literature reviews may be incomplete, thus undermining the fundamental basis of any research study. In addition, inaccuracies in referencing can jeopardize academic integrity and diminish the credibility of research findings. These gaps hinder the production of high-quality, evidence-based research and impede the dissemination and application of research findings in clinical practice. To address these gaps, targeted interventions are essential. These interventions should include workshops that focus on research methodologies and database navigation. In addition, mentoring programs that pair inexperienced researchers with experienced mentors should be established, and partnerships with research-intensive institutions should be formed to provide additional training and resources. By implementing these specialized educational initiatives and promoting international collaborations, tangible improvements in research capacity and output can be achieved in the region.

Despite the disappointing results, they are consistent with the educational qualifications of the faculty members. The majority of faculty members possess only bachelor's or master's degrees, suggesting limited involvement in research. This finding aligns with a study conducted by Candela et al. (2016), which emphasizes the critical role of education in enhancing research skills among nurses. The study found that nurses who received relevant clinical research training, had prior research participation experience, and possessed consultancy experience exhibited higher levels of knowledge and more positive attitudes toward research [23]. Chen et al. (2006) also emphasized the significance of education as a fundamental investment factor that shapes the research productivity of faculty members. The study highlighted the importance of tenure, promotion, and salary increments as incentives for engaging in scholarly activities [24]. Therefore, education not only equips nurses with essential skills and knowledge but also enhances their motivation and effectiveness in conducting research.

Regarding the participants' knowledge levels in our study, nearly half of the nursing faculty members exhibited low levels of knowledge, while approximately one-third displayed moderate levels of knowledge. A smaller subset, comprising 11.6% of the participants, demonstrated high levels of research knowledge. These findings suggest that obstacles such as insufficient financial support and limited access to updated platforms may hinder the advancement of knowledge among faculty members, impeding their ability to engage in scientific research. Similarly, Darawad et al. (2018) found that nursing faculty members in a Saudi public university held moderately negative attitudes toward research. They found that organizational barriers outweighed individual barriers. Despite these challenges, participants expressed a relatively strong intention to participate in research activities. To enhance the research productivity of nursing faculty members in Saudi Arabia, it is crucial to address obstacles, such as insufficient financial support, teaching overload, and limited access to information resources [11].

The study also revealed a highly significant association (p < 0.004) between the level of education and participants' knowledge levels. This indicates that individuals with higher levels of education tend to possess greater proficiency in terms of knowledge. In addition, a significant association (p < 0.011) was identified between years of experience and knowledge level. This suggests that participants with more experience tend to exhibit higher levels of knowledge. These findings highlight the importance of both educational attainment and professional experience in shaping the knowledge base of nursing faculty members. Marzilli's (2016) study also supports this correlation between education and knowledge level. It found that participants, primarily with an MSN education, scored lower on the knowledge subscale of the Nurses' Cultural Competence Scale (NCCS) compared to other subscales. This suggests a potential difference in knowledge proficiency among faculty members, regardless of their educational attainment [25].

By contrast, Stichler et al. (2011) conducted a study that showed the possession of a doctoral degree accounted for only a small amount of the variability in knowledge and skills related to evidence-based practice (EBP). The hierarchical multiple regression analysis indicated that none of the predictor variables achieved statistical significance in this regard. These findings challenge the assumption that individuals with a PhD would have higher levels of knowledge in teaching EBP. The type of degree held did not consistently correlate with superior knowledge in EBP [26].

Strengths and limitations and future research

While the study provides valuable insights into the research knowledge of nursing faculty members at the University of Raparin, it is important to acknowledge several limitations. First, the study is limited to a specific institution, which may limit the generalizability of the findings to other nursing education programs. Second, the use of a cross-sectional design only captures knowledge levels at a single point in time and does not allow for tracking changes or trends over time. Relying on self-reported data from participants introduces the possibility of response biases or inaccuracies in reporting their knowledge levels. Finally, one significant limitation of this study is its small sample size, which may affect the generalizability of the findings. Future research should aim to involve a larger and more diverse sample from multiple institutions in order to improve the generalizability of findings. Longitudinal designs can be utilized to monitor changes in research knowledge over time. In addition, it is crucial to conduct comprehensive data collection on factors that impact research knowledge. Despite these limitations, the study possesses several strengths that contribute to its significance in understanding the research knowledge of nursing faculty members. First, the study is highly relevant to nursing education as it addresses a crucial aspect of faculty development necessary for advancing nursing research and evidence-based practice. Moreover, the study's conclusions provide actionable recommendations for improving research literacy among nursing faculty, potentially leading to tangible improvements in research quality and practice within the field. Finally, the study's identification of knowledge gaps and its recommendations for future action serve as a roadmap for implementing comprehensive training programs and support structures to address these deficiencies and foster a culture of research excellence among nurse educators.

## Conclusions

The results of the study highlight a significant lack of research knowledge among nursing faculty members. The widespread deficiency among the majority of faculty members emphasizes the need for targeted interventions and educational initiatives in the nursing education curriculum. It is crucial to bridge this knowledge gap to promote faculty members' professional development and advance nursing research and evidence-based practice. Going forward, it is essential to prioritize the implementation of comprehensive training programs and support systems to empower nurse educators to enhance their research literacy. This will ultimately improve the quality of research in the field of nursing education.

## Appendices

Title: Assessment of Nursing Faculty Members' Knowledge toward Research: A Cross-Sectional Study

Name of Researchers

Sirwan Khalid Ahmed, Ribwar Arsalan Mohammed, Kochr Ali Mahmood, Omaid Saber Abdullah, Hazhar Talaat Abubaker Blbas, Araz Qadir Abdalla, Mohammed Khdhir Hamad, Mona Gamal Mohammed

Questionnaires

Dear Colleague,

The following questionnaire is designed to help our researchers to “Assessment of Nursing Faculty Members' Knowledge toward Research: A Cross-Sectional Study”. All the data in this questionnaire will be used only for research purposes. No personal data are required. We would be grateful if you would help us by completing the form. By agreeing to complete this form, you are giving us permission to use the data in our research project.  

Part one: Demographic of respondents

1. Age/ Years

2. Gender

a. Male

b. Female

3. Marital Status

a. Single

b. Married

4. What is your current degree

Bachelor                            

Master

PhD

5. Year of experience as a faculty member

6. Participated in research writing and publication courses

A. Yes

B. No                                      

Part two: Assessment of nursing faculty members' knowledge of research.

This part of the questionnaire aims to assess your knowledge of medical research by asking about some basic concepts, please choose the first answer that comes to your mind, and feel free to choose “I don’t know” if you don’t.

1. Which software is used for reference of science articles?

A) SPSS           B) Access                   C) Concept map                     D) End note                   E) I don’t know

2. What is the variable scale of blood type?

A) Nominal      B) Relative                    C) Distance                  D) Serial                          E) I don’t know

3. Which way of writing reference is approved for writing general medical dissertations and most medical journals? 

A) Vancouver           B) Harvard              C) None                        D) Chicago                      E) I don’t know

4. What is the definition of Medline?

A) The first and best-known on-line medical journals

B) Association for informed medical providers

C) Print format chosen for medical

D) Medical database

E) I don’t know

5. In which part of the article do you talk about the study's limitations? 

A) Acknowledgment    B) Methods and Materials   C) Discussion         D) Introduction               E) I don’t know

6. Which of the following types of research sees sample loss more commonly

A) Clinical trial        B) Cross-sectional                  C) Case-control        D) Cohort                        E) I don’t know

7. Which item is not part of a scientific original paper?

A) Discussion        B) Introduction                          C) Letters to the Editor       D) Methods and Materials

E) I don’t know
